# RETRACTED: Sudha et al. Triazole Modified Tetraiodothyroacetic Acid Conjugated to Polyethylene Glycol, a Thyrointegrin α_v_β_3_ Antagonist as a Radio- and Chemo-Sensitizer in Pancreatic Cancer. *Biomedicines* 2022, *10*, 795

**DOI:** 10.3390/biomedicines13112641

**Published:** 2025-10-28

**Authors:** Thangirala Sudha, Kavitha Godugu, Gennadi V. Glinsky, Shaker A. Mousa

**Affiliations:** 1 The Pharmaceutical Research Institute, Albany College of Pharmacy and Health Sciences, Rensselaer, NY 12144, USA; sudha.thangirala@acphs.edu (T.S.); kavitha.godugu@acphs.edu (K.G.); 2 Institute of Engineering in Medicine, University of California, San Diego, CA 92093, USA; gglinskii@ucsd.edu

The journal retracts the article “Triazole Modified Tetraiodothyroacetic Acid Conjugated to Polyethylene Glycol, a Thyrointegrin α_v_β_3_ Antagonist as a Radio- and Chemo-Sensitizer in Pancreatic Cancer” [1] cited above.

Following publication, concerns were brought to the attention of the Editorial Office regarding the integrity of and images presented in this publication [1].

Adhering to our standard procedure, an investigation was conducted by the Editorial Office and Editorial Board that identified indications of inappropriate editing and similarities among the subfigures presented in Figure 3. While the authors collaborated within this process, the raw material meeting the journal’s requirements for original images could not be provided for Editorial Board evaluation. Consequently, the Editorial Board has lost confidence in the reliability of the findings and has decided to retract this publication, as per MDPI’s retraction policy (https://www.mdpi.com/ethics#_bookmark30).

This retraction was approved by the Editor-in-Chief of the journal *Biomedicines*. The authors did not provide a comment on this decision.
